# Epidemiology of Stevens-Johnson syndrome and toxic epidermal necrolysis in the United States and factors predictive of outcome

**DOI:** 10.1016/j.jdin.2023.06.014

**Published:** 2023-07-11

**Authors:** Paul Wasuwanich, Joshua M. So, Teja S. Chakrala, Jinghua Chen, Kiran Motaparthi

**Affiliations:** a University of Florida College of Medicine, Gainesville, Florida; b Department of Internal Medicine, University of Florida College of Medicine, Gainesville, Florida; c Department of Ophthalmology, University of Florida College of Medicine, Gainesville, Florida; d Department of Dermatology, University of Florida College of Medicine, Gainesville, Florida

**Keywords:** drug eruptions, inpatient dermatology, risk factors, severe cutaneous adverse reactions, Stevens-Johnson syndrome, toxic epidermal necrolysis

## Abstract

**Background:**

Stevens-Johnson syndrome (SJS), toxic epidermal necrolysis (TEN), and SJS-TEN overlap syndrome are rare severe cutaneous adverse reactions associated with high mortality.

**Objectives:**

To estimate incidence and describe trends of SJS/TEN hospitalizations in the United States and to describe the clinical, demographic, and geographic characteristics of affected patients and risk factors for mortality.

**Methods:**

We utilized hospitalization data from the 2010 to 2020 National Inpatient Sample. SJS, SJS-TEN overlap syndrome, and TEN were identified by International Classification of Diseases, 9th Revision and International Classification of Diseases, 10th Revision codes and analyzed by logistic regression.

**Results:**

We identified 51,040 hospitalizations involving SJS/TEN. Amog those, 37,283 (73.0%) were for SJS only, 7818 (15.3%) were for SJS-TEN overlap syndrome, and 7160 (14.0%) were for TEN only. Overall, SJS/TEN hospitalization rates declined over time, 2010 to 2020 (*P* < .05). Mortality rates of the SJS group, SJS-TEN overlap syndrome group, and TEN group were 5.4%, 14.4%, and 15.3%, respectively. Increasing age, chronic kidney disease, pneumonia, sepsis, and malignant neoplasm were all significantly associated with increased odds of mortality (*P* < .05). Non-Hispanic White racial/ethnic identification was associated with decreased odds of mortality (*P* < .05).

**Limitations:**

Lack of standardization for diagnostic criteria.

**Conclusions:**

Risk factors identified in this study lay the groundwork for improvement in SJS/TEN mortality prediction scoring.

**Capsule Summary**

• Stevens-Johnson syndrome and toxic epidermal necrolysis have high mortality rates with risk factors for mortality identified in Severity-of-Illness Score for Toxic Epidermal Necrolysis score.
• We reaffirm most risk factors within Severity-of-Illness Score for Toxic Epidermal Necrolysis but propose improvements: utilizing age as a continuous variable and including chronic kidney disease, pneumonia, sepsis, and race/ethnicity as independent risk factors.

## Introduction

Stevens-Johnson syndrome (SJS), toxic epidermal necrolysis (TEN), and SJS-TEN overlap syndrome are rare but severe cutaneous adverse reactions, typically caused by drug exposure.^1^^,^^2^ SJS/TEN includes SJS, SJS-TEN overlap syndrome, and TEN; they are generally considered a spectrum of a single disease entity, and are defined as polymorphic lesions involving <10%, 10% to 30%, and >30% of the body surface area (BSA), respectively.^3^

SJS/TEN is a disease spectrum with high morbidity and mortality; previous studies have reported mortality rates for SJS to be around 19.4% to 29% and for TEN to be around 14.8% to 48%.^4^, ^5^, ^6^ Known risk factors associated with mortality include age older than 40 years, the presence of associated malignancy, BSA involvement >10%, serum bicarbonate <20 mmol/L, serum urea nitrogen >10 mmol/L, serum glucose >14 mmol/L, heart rate >120 beats per minute, and chronic kidney disease.^7^^,^^8^

As SJS/TEN is relatively rare, studies are often limited by sample size. Updated epidemiologic data for SJS/TEN are lacking in the United States. Using the National Inpatient Sample (NIS), we aim to estimate the incidence of SJS/TEN, describe trends of SJS/TEN hospitalizations in the United States, and to analyze the clinical, demographic, and geographic characteristics of affected patients and risk factors for mortality between 2010 and 2020.

## Materials and methods

### Study population

Following approval from the Institutional Review Board of the University of Florida, we utilized data from NIS, the largest all-payer database on hospital inpatient stays in the United States. NIS is a part of the Healthcare Cost and Utilization Project.^9^^,^^10^ A 5-year interval from September 1st, 2015 to December 31st, 2020 was selected for full analysis due to the consistent use of the International Classification of Diseases, 10th Revision (ICD-10) diagnosis codes which were implemented on September 1st, 2015 and replaced the International Classification of Diseases, 9th Revision (ICD-9) diagnosis codes. The interval between January 1st, 2010 and September 1st, 2015 was limited in analysis due to the differences in International Classification of Diseases code versions.

### Data extraction

We queried the NIS database for cases of SJS, SJS-TEN overlap syndrome, and TEN. SJS was identified by ICD-9 diagnosis code 695.13 and by ICD-10 diagnosis code L51.1. SJS-TEN overlap syndrome was identified by ICD-9 diagnosis code 695.14 and by ICD-10 diagnosis code L51.3. TEN was identified by ICD-9 diagnosis code 695.15 and by ICD-10 diagnosis code L51.2. SJS, SJS-TEN overlap syndrome, and TEN cases with length of hospital stay less than 3 days were excluded.^11^

We extracted demographic and geographic data including age, sex, race/ethnicity, household income quartile, hospital charge (in US dollars), insurance status, region of hospital, size of hospital, hospital ownership, and type of hospital (rural, urban nonteaching, or urban teaching). The following clinical data were obtained: length of hospital stay, all-cause mortality, diabetes mellitus type I/II, obesity, inborn errors of urea cycle metabolism, corneal ulcer, conjunctivitis, blindness, chronic kidney disease, solid organ transplant status, hepatitis B, hepatitis C, HIV infection, mycoplasma infection, herpes simplex virus infection, herpes zoster infection, tuberculosis, skin/subcutaneous infections, pneumonia, sepsis, malignant neoplasm, cutaneous autoimmune disease (lupus erythematosus, dermatomyositis, pemphigoid, or pemphigus), and history of intravenous drug use (IVDU). History of IVDU was estimated and determined indirectly. Hospitalizations with the diagnosis of dependence on drugs commonly injected intravenously including opioids, sedatives, amphetamines, hallucinogens, or combinations of these were classified as being associated with IVDU.

### Statistical analysis

Hospitalization rates were analyzed for trends between 2010 and 2020 using Poisson regression. Case-fatality rates were calculated as the percentage of deaths that resulted from all hospitalizations for a given disease. The nonnormal data were summarized using the median and interquartile range (IQR) and compared using the Mann-Whitney *U* test. Frequencies were compared using the χ^2^ test. Risk factors for mortality among SJS/TEN hospitalizations were analyzed by logistic regression in both univariable (crude) and multivariable (adjusted) models. The dichotomous outcomes were alive at discharge versus death; results were reported by odds ratio (OR) with 95% confidence intervals (CIs). ORs were derived from exponentiated beta logistic regression coefficients. For a continuous variable, the OR represents the odds for each unit increase of that variable. The main analysis involved the entire SJS/TEN spectrum; subsequent subgroup analyses were conducted for SJS, SJS-TEN overlap syndrome, and TEN subgroups and presented in the Supplementary Materials, available via Mendeley at https://doi.org/10.17632/wr4gcnnftn.1.

All results reported were weighted, using discharge weights provided by Healthcare Cost and Utilization Project to present the true number of hospitalizations. Results for a category that contained greater than zero but 10 or fewer hospitalizations were displayed as ≤10 due to the data use privacy policy of Healthcare Cost and Utilization Project. Missing data were assumed to be missing at random.

Statistical significance was defined as *P* < .05. The Benjamin-Hochberg procedure was applied to control for multiple comparisons with selected false discovery rate of 5%. Statistical calculations were performed using R program.

## Results

**Fig 1:**
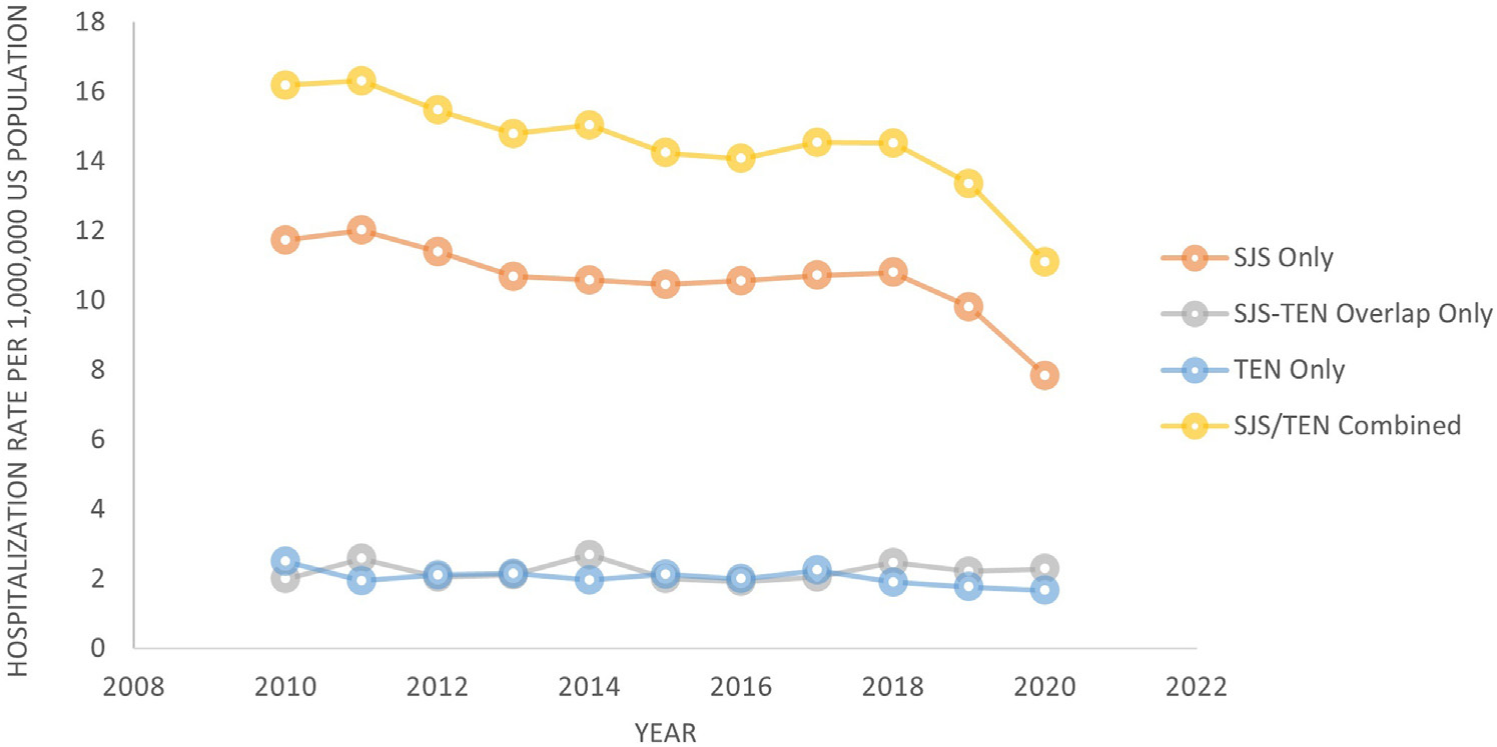
Hospitalization rate over time for Stevens-Johnson syndrome (SJS) and toxic epidermal necrolysis (TEN), 2010 to 2020. SJS/TEN combined category includes SJS, SJS-TEN overlap syndrome, and TEN. *SJS*, Stevens-Johnson syndrome; *TEN*, toxic epidermal necrolysis.

**Fig 2:**
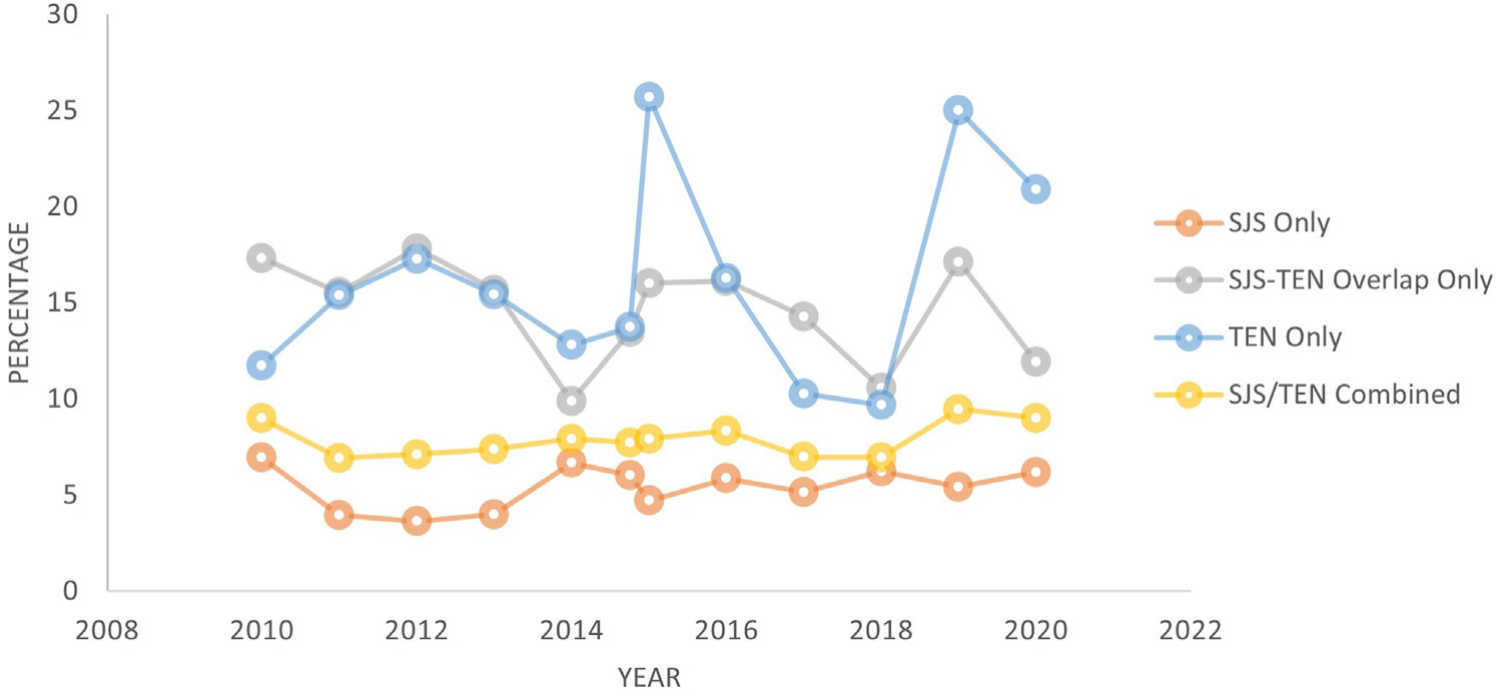
Mortality rates over time for Stevens-Johnson syndrome (SJS) and toxic epidermal necrolysis (TEN), 2010 and 2020. SJS/TEN combined category includes SJS, SJS-TEN overlap syndrome, and TEN. *SJS*, Stevens-Johnson syndrome; *TEN*, toxic epidermal necrolysis.

Out of 392,302,031 estimated hospitalizations in the United States between 2010 and 2020, we identified 51,040 (0.1%) hospitalizations for SJS/TEN. Among those, 37,283 (73.0%) were for SJS, 7818 (15.3%) were for SJS-TEN overlap syndrome, and 7160 (14.0%) were for TEN. Between 2010 and 2020, there was an average of 4640 hospitalizations for SJS/TEN per year in the United States. The hospitalization rate of SJS, SJS-TEN overlap syndrome, and TEN cases over time are displayed in Fig 1. From 2010 to 2020, hospitalization rates of SJS/TEN decreased over time (95% CI: 0.97-0.98; *P* < .001). Among the 51,040 patients hospitalized for SJS/TEN, 4012 (7.9%) died. Mortality rates in the SJS group, SJS-TEN overlap syndrome group, and TEN group were 5.4%, 14.4%, and 15.3%, respectively. Mortality rates over time are displayed in Fig 2.

In the 2015 to 2020 period, 23,265 patients were hospitalized for SJS/TEN. Among those, 13,305 (57.2%) were female, and the median age was 58 years (IQR: 39-71). The majority of patients hospitalized were non-Hispanic white (*N* = 12,265; 52.7%) and located in the Southern region of the United States (*N* = 10,205; 43.9%). Among the 23,265 patients hospitalized for SJS/TEN between 2015 and 2020, most were in the 0 to 25th percentile for household income (*N* = 7875; 33.8%) (Table I). We found that patients who died were older, with a median age of 64 years (IQR: 52-75) versus 57 years (IQR: 37-71) (*P* < .001). Additionally, the group of patients that died of SJS/TEN contained a greater proportion of non-Hispanic Black patients, 615 (32.7%) versus 5265 (24.6%) (*P* < .001). Urban teaching hospitals were a more frequent type of hospital among patients who died, 1605 (85.4%) versus 16,585 (77.6%) (*P* = .002). Additionally, medicare was a more frequent payer among patients who died, 1010 (53.7%) versus 9815 (45.9%) (*P* = .009). The median total hospital charge was significantly higher among patients who died than among patients, $207,321 (IQR: $90,538-$400,936) versus $54,931 (IQR: $27,895-$120,021) (*P* < .001). Sex, region of hospitalization, hospital bed size, hospital ownership, and household income quartile were not significantly associated with mortality. Further details are summarized in Table I. Supplementary Tables I-III, available via Mendeley at https://doi.org/10.17632/wr4gcnnftn.1 display these data for SJS, SJS-TEN overlap syndrome, and TEN subgroups when analyzed separately.

**Table I:** Demographic, geographic, and social characteristics of Stevens-Johnson syndrome and toxic epidermal necrolysis hospitalizations by outcome (mortality)

Characteristics	SJS/TEN survived	SJS/TEN died	*P* value
Number of hospitalizations, *N*	21,385	1880	
Age, y, median (IQR)	57 (37-71)	64 (52-75)	**<.001**∗
Sex, *N* (%)			**.032**
Male	9255 (43.3)	705 (37.5)	
Female	12,130 (56.7)	1175 (62.5)	
Race/ethnicity, *N* (%)			**<.001**∗
Non-Hispanic White	11,465 (53.6)	800 (42.6)	
Non-Hispanic Black	5265 (24.6)	615 (32.7)	
Hispanic	2025 (9.5)	135 (7.2)	
Asian or Pacific Islander	935 (4.4)	105 (5.6)	
Native American	140 (0.7)	15 (0.8)	
Other/unknown	1555 (7.3)	210 (11.2)	
Region of hospital, *N* (%)			.208
Northeast	3525 (16.5)	260 (13.8)	
Midwest	4290 (20.1)	370 (19.7)	
South	9290 (43.4)	915 (48.7)	
West	4280 (20.0)	335 (17.8)	
Type of hospital, *N* (%)			**.002**∗
Rural	1360 (6.4)	80 (4.3)	
Urban nonteaching	3440 (16.1)	195 (10.4)	
Urban teaching	16,585 (77.6)	1605 (85.4)	
Hospital bed size, *N* (%)			.359
Small	3430 (16.0)	255 (13.6)	
Medium	6015 (28.1)	515 (27.4)	
Large	11,940 (55.8)	1110 (59.0)	
Hospital ownership, *N* (%)			.223
Government, nonfederal	3065 (14.3)	300 (16.0)	
Private, not-profit	14,975 (70.0)	1345 (71.5)	
Private, invest-own	3345 (15.6)	235 (12.5)	
Expected primary payer, *N* (%)			**.009**∗
Medicare	9815 (45.9)	1010 (53.7)	
Medicaid	4335 (20.3)	310 (16.5)	
Private insurance	5865 (27.4)	465 (24.7)	
Self-pay	650 (3.0)	20 (1.1)	
No charge	90 (0.4)	0 (0)	
Other	630 (2.9)	75 (4.0)	
Household income quartile, *N* (%)			.650
0-25th percentile	7185 (33.6)	690 (36.7)	
26th-50th percentile	5330 (24.9)	460 (24.5)	
51st-75th percentile	4535 (21.2)	400 (21.3)	
76th-100th percentile	3920 (18.3)	310 (16.5)	
Total hospital charge, USD, median (IQR)	54,931 (27,895-120,021)	207,321 (90,538-400,936)	**<.001**∗

National Inpatient Sample, Healthcare Cost and Utilization Project, 2015-2020.

*P* values of statistical significance (*P* < .05) were bolded.

*IQR*, Interquartile Range; *SJS*, Stevens-Johnson syndrome; *TEN*, toxic epidermal necrolysis; *USD*, United States dollar.

∗ *P* values that were significant after controlling for multiple comparisons by Benjamin-Hochberg procedure.

We analyzed clinical features associated with increased mortality in patients with SJS/TEN. The median hospital length of stay was found to be significantly greater among patients who died compared to patients who survived, 11 days (IQR: 6-21) versus 7 days (IQR: 4-12) (*P* < .001), respectively. Diabetes mellitus [670 (35.6%) versus 6125 (28.6%) (*P* = .004)], inborn errors of urea cycle metabolism [20 (1.1%) vs 45 (0.2%) (*P* = .003)], chronic kidney disease [820 (43.6%) vs 5170 (24.2%) (*P* < .001)], pneumonia [650 (34.6%) vs 2835 (13.3%) (*P* < .001)], sepsis [1365 (72.6%) vs 4405 (20.6%) (*P* < .001)], and the presence of a malignant neoplasm [415 (22.1%) vs 2105 (9.8%) (*P* < .001)] were more frequent among patients who died of SJS/TEN compared to those who survived, respectively. In contrast, conjunctivitis [50 (2.7%) vs 1685 (7.9%) (*P* < .001)], mycoplasma infection [≤10 vs 555 (2.6%) (*P* = .005)], and IVDU [30 (1.6%) vs 910 (4.3%) (*P* = .012)] were less frequent among patients who died of SJS/TEN compared to patients who survived, respectively. Obesity, blindness, corneal ulcer, solid organ transplantation, hepatitis B or C, HIV, herpes simplex virus or herpes zoster infection, tuberculosis, skin/subcutaneous infections, and cutaneous autoimmune disease were not associated with mortality. Additional details are summarized in Table II. Supplementary Tables IV-VI, available via Mendeley at https://doi.org/10.17632/wr4gcnnftn.1 display these data for SJS, SJS-TEN overlap syndrome, and TEN subgroups when analyzed separately.

**Table II:** Clinical characteristics of Stevens-Johnson syndrome and toxic epidermal necrolysis hospitalizations by mortality outcome

Characteristics	SJS/TEN total	SJS/TEN survived	SJS/TEN died	*P* value
Number of hospitalizations, *N*	23,265	21,385	1880	
Length of stay, d, median (IQR)	7 (4-12)	7 (4-12)	11 (6-21)	**<.001**∗
Metabolic disorders, *N* (%)				
Diabetes	6795 (29.2)	6125 (28.6)	670 (35.6)	**.004**∗
Obesity	4250 (18.3)	3955 (18.5)	295 (15.7)	.175
Urea cycle metabolism disorder	65 (0.3)	45 (0.2)	20 (1.1)	**.003**∗
Ophthalmic disorders, *N* (%)				
Blindness	545 (2.3)	525 (2.5)	20 (1.1)	.087
Corneal ulcer	95 (0.4)	90 (0.4)	≤10	.651
Conjunctivitis	1735 (7.5)	1685 (7.9)	50 (2.7)	**<.001**∗
Organ dysfunction, *N* (%)				
Chronic kidney disease	5990 (25.7)	5170 (24.2)	820 (43.6)	**<.001**∗
Solid organ transplant recipient	180 (0.8)	155 (0.7)	25 (1.3)	.200
Infections, *N* (%)				
Hepatitis B or C	665 (2.9)	610 (2.9)	55 (2.9)	.916
HIV positive	475 (2.0)	430 (2.0)	45 (2.4)	.617
Mycoplasma infection	560 (2.4)	555 (2.6)	≤10	**.005**∗
Herpes simplex virus infection	605 (2.6)	535 (2.5)	70 (3.7)	.155
Herpes zoster	150 (0.6)	130 (0.6)	20 (1.1)	.291
Tuberculosis	50 (0.2)	40 (0.2)	≤10	.167
Skin/subcutaneous Infections	3415 (14.7)	3180 (14.9)	235 (12.5)	.211
Pneumonia	3485 (15.0)	2835 (13.3)	650 (34.6)	**<.001**∗
Sepsis	5770 (24.8)	4405 (20.6)	1365 (72.6)	**<.001**∗
Other, *N* (%)				
Malignant neoplasm	2520 (10.8)	2105 (9.8)	415 (22.1)	**<.001**∗
Cutaneous autoimmune disease	1310 (5.6)	1245 (5.8)	65 (3.5)	.056
Intravenous drug use	940 (4.0)	910 (4.3)	30 (1.6)	**.012**∗

National Inpatient Sample, Healthcare Cost and Utilization Project, 2015-2020.

*P* values of statistical significance (*P* < .05) were bolded.

*SJS*, Stevens-Johnson syndrome; *TEN*, toxic epidermal necrolysis.

∗ *P* values that were significant after controlling for multiple comparisons by Benjamin-Hochberg procedure.

Using logistic regression, we investigated risk factors potentially associated with increased mortality in the SJS/TEN cohort. In the univariable (crude) model, increasing age, diabetes mellitus, inborn errors of urea cycle metabolism, chronic kidney disease, pneumonia, sepsis, and malignant neoplasm were all significantly associated with increased odds of mortality. Non-Hispanic White race/ethnicity and conjunctivitis were significantly associated with decreased odds of mortality. In the multivariable (adjusted) model, increasing age (OR = 1.01; 95% CI = 1.01-1.02; *P* < .001) with risk of mortality increasing yearly starting at 25 years, chronic kidney disease (OR = 1.52; 95% CI = 1.16-2.00; *P* = .002), pneumonia (OR = 1.76; 95% CI = 1.32-2.33; *P* < .001), sepsis (OR = 8.21; 95% CI = 6.28-10.73; *P* < .001), and malignant neoplasm (OR = 3.11; 95% CI = 2.29-4.24; *P* < .001) retained significantly associated increased odds of mortality. Similarly, non-Hispanic White race/ethnicity (OR = 0.71; 95% CI = 0.56-0.92; *P* = .009) remained significantly associated with decreased odds of mortality. In contrast, diabetes mellitus, inborn errors of urea cycle metabolism, and conjunctivitis did not retain significant associations with odds of mortality in the multivariable model. Sex, household income quartile, obesity, blindness, corneal ulcer, solid organ transplantation, hepatitis B, C, or HIV infection, mycoplasma infection, herpes simplex virus infection, herpes zoster infection, tuberculosis, skin/subcutaneous infections, cutaneous autoimmune disease, and IVDU were not identified as risk factors based on logistic regression. Further details are summarized in Table III. Supplementary Tables VII-IX, available via Mendeley at https://doi.org/10.17632/wr4gcnnftn.1 reflect these data for SJS, SJS-TEN overlap, and TEN subgroups when analyzed separately.

**Table III:** Risk factors for mortality Stevens-Johnson syndrome and toxic epidermal necrolysis

Risk factors	Comparison	Crude odds ratio (95% CI)	Crude *P* value	Adjusted odds ratio (95% CI)	Adjusted *P* value
Demographics					
Age	High (vs low)—quant.	1.02 (1.02-1.03)	**<.001**∗	1.01 (1.01-1.02)	**<.001**∗
Sex	Female (vs male)	1.27 (1.02-1.58)	**.032**	1.38 (1.07-1.77)	**.014**
Race/ethnicity	White (vs other)	0.65 (0.52-0.81)	**<.001**∗	0.71 (0.56-0.92)	**.009**∗
Household income quartile	High (vs low)—quant.	0.94 (0.86-1.04)	.245	0.99 (0.89-1.11)	.896
Metabolic disorders					
Diabetes	Yes (vs no)	1.38 (1.10-1.72)	**.005**∗	0.96 (0.73-1.26)	.769
Obesity	Yes (vs no)	0.82 (0.61-1.09)	.176	0.73 (0.52-1.01)	.060
Urea cycle metabolism disorder	Yes (vs no)	5.09 (1.56-16.63)	**.007**∗	4.40 (0.67-28.89)	.122
Ophthalmic disorders					
Blindness	Yes (vs no)	0.43 (0.16-1.17)	.097	0.58 (0.19-1.78)	.339
Corneal ulcer	Yes (vs no)	0.63 (0.08-4.74)	.654	0.78 (0.07-8.89)	.842
Conjunctivitis	Yes (vs no)	0.32 (0.17-0.61)	**<.001**∗	0.63 (0.30-1.31)	.217
Organ dysfunction					
Chronic kidney disease	Yes (vs no)	2.42 (1.95-3.01)	**<.001**∗	1.52 (1.16-2.00)	**.002**∗
Solid organ transplant recipient	Yes (vs no)	1.84 (0.71-4.77)	.207	1.04 (0.33-3.28)	.952
Infections					
Hepatitis B or C	Yes (vs no)	1.03 (0.55-1.94)	.916	1.01 (0.45-2.24)	.982
HIV positive	Yes (vs no)	1.19 (0.60-2.39)	.617	1.16 (0.53-2.55)	.717
Mycoplasma infection	Yes (vs no)	0.10 (0.01-0.72)	**.022**	0.29 (0.03-2.46)	.254
Herpes simplex virus infection	Yes (vs no)	1.51 (0.85-2.66)	.157	1.66 (0.86-3.21)	.130
Herpes zoster	Yes (vs no)	1.76 (0.61-5.06)	.297	0.66 (0.18-2.36)	.520
Tuberculosis	Yes (vs no)	2.85 (0.60-13.48)	.186	2.73 (0.62-12.01)	.185
Skin/subcutaneous infections	Yes (vs no)	0.82 (0.59-1.12)	.211	0.66 (0.46-0.96)	**.028**
Pneumonia	Yes (vs no)	3.45 (2.74-4.35)	**<.001**∗	1.76 (1.32-2.33)	**<.001**∗
Sepsis	Yes (vs no)	10.20 (8.04-12.95)	**<.001**∗	8.21 (6.28-10.73)	**<.001**∗
Other					
Malignant neoplasm	Yes (vs no)	2.59 (1.99-3.37)	**<.001**∗	2.38 (1.73-3.28)	**<.001**∗
Cutaneous autoimmune disease	Yes (vs no)	0.58 (0.33-1.02)	.059	0.47 (0.25-0.91)	**.025**
Intravenous drug use	Yes (vs no)	0.36 (0.16-0.83)	**.016**	0.47 (0.20-1.13)	.092

National Inpatient Sample, Healthcare Cost and Utilization Project, 2015-2020.

*P* values of statistical significance (*P* < .05) were bolded.

∗ *P* values that were significant after controlling for multiple comparisons by Benjamin-Hochberg procedure.

Supplementary Fig 1, available via Mendeley at https://doi.org/10.17632/wr4gcnnftn.1 highlights the inverse correlation between mortality due to SJS/TEN and household income quartile. Supplementary Fig 2, available via Mendeley at https://doi.org/10.17632/wr4gcnnftn.1 demonstrates the direct correlation between mortality and hospital bed size. Supplementary Fig 3, available via Mendeley at https://doi.org/10.17632/wr4gcnnftn.1 highlights disparities in mortality due to SJS/TEN across races/ethnicities.

## Discussion

Our study includes the largest single cohort of patients with SJS/TEN, with over 50,000 hospitalizations across 11 years. A previous nationwide study by Hsu et al investigated the morbidity and mortality of SJS/TEN in the United States but was limited to data from 2009 to 2012 and did not investigate SJS-TEN overlap syndrome in detail.^5^

The mortality rates of 5.4% and 15.3% for SJS and TEN, respectively, are similar to those previously reported.^5^ However, Hsu et al^5^ reported a mortality rate of 19.4% for the SJS-TEN overlap syndrome subgroup, compared to 14.4% in our larger study. In the SJS/TEN subgroups, we identified increased mortality with increased BSA involvement, but this increase was not linear, increasing greatly between SJS and SJS-TEN overlap syndrome; in contrast, mortality rates are similar between SJS-TEN overlap syndrome and TEN. Given these prognostic implications, it is important to clinically differentiate SJS from SJS-TEN overlap syndrome and TEN.

There was a notable female predominance in the SJS/TEN cohort (57.2%). In the TEN subgroup, the female predominance was greater (58.6%, Supplementary Table II). Historically, the reported female to male ratio of patients with SJS/TEN has ranged between 3:2 and 2:1;^2^^,^^12^ however, in our study, this ratio is closer to 4:3 for SJS/TEN overall and 3:2 for the TEN subgroup.

In 2000, Bastuji-Garin et al^7^ developed the Severity-of-Illness Score for Toxic Epidermal Necrolysis (SCORTEN) system, based on a sample of 165 patients and identified 7 independent risk factors of mortality (age ≥40 years, heart rate ≥120 beats per min, cancer/hematologic malignancy, BSA detached ≥10% at day 1, serum blood urea nitrogen [BUN] >10 mmol/L, serum bicarbonate <20 mmol/L, and serum glucose >14 mmol/L). In our study, increasing age, chronic kidney disease, and the presence of a malignant neoplasm were associated with increased odds of mortality. We propose a gradient model of age-related risk, to reflect the risk of mortality increasing with each additional year of age starting at age 25, as opposed to a binary cutoff of ≥40 years as in the SCORTEN model^7^ or ≥50 years as in the ABCD-10 (age, bicarbonate, cancer, dialysis, 10% body surface area risk) model.^13^ We were not able investigate serum BUN. While chronic kidney disease often results in increased serum BUN, chronic kidney disease and BUN may both serve as independent risk factors for mortality in SJS/TEN.^8^ Conversely, we investigated inborn errors in urea cycle metabolism, which are associated with decreased levels of BUN^14^ but have not previously been investigated in the SJS/TEN. Inborn errors in urea cycle metabolism may be associated with mortality in SJS/TEN, but the sample size of those conditions is too small to be conclusive.

We found associations between outcomes and racial/ethnic disparities as well as income disparities. Non-Hispanic White race/ethnicity was associated with decreased odds of mortality. It is unclear whether there are true biological differences among patients of different races/ethnicities relevant to SJS/TEN; however, it is possible that patients with darker skin types may experience a delay in diagnosis or treatment of SJS/TEN due to limited education on dermatologic diseases in skin of color.^15^ While the association of household income quartile with mortality in SJS/TEN was insignificant, there was a trend of increasing mortality with decreasing household income quartile (Supplementary Fig 1).

Pneumonia and sepsis are independent risk factors that were associated with increased odds of mortality in the SJS/TEN population. Hsu et al reported tuberculosis as a strong risk factor for mortality^5^; however, in our study, tuberculosis was rare (50 cases) within the SJS/TEN cohort and not found to be associated with mortality. Hsu et al used data from 2009 to 2012 while we primarily used data from 2015 to 2020. The difference in these results may reflect the declining incidence of tuberculosis in the United States over time.

Autoimmune cutaneous diseases, particularly lupus erythematosus, are associated with the development of SJS/TEN.^16^^,^^17^ In a recent study, Frey et al reported that lupus erythematosus may be associated with developing SJS/TEN (OR = 16.00, 95% CI = 1.79-143.15).^17^ However, based on our large sample, autoimmune cutaneous diseases are not associated with increased mortality due to SJS/TEN.

A limitation of this study was the lack of standardization for diagnostic criteria in the NIS database. SJS-TEN overlap syndrome is likely to be underdiagnosed by nondermatologists, leading to a potential underestimation of its true incidence. As there was no specific diagnostic code for IVDU, the reported number of IVDU cases is potentially an overestimation. A potential for miscoding also exists; we presumed that any miscoded diagnoses by physicians would be randomly distributed throughout the database and therefore would not result in statistically significant differences between subgroups. Lastly, we did not apply a comorbidity index to our results.

## Conclusion

While SJS/TEN is relatively rare, it is associated with high mortality and directly correlates with BSA involved, increasing from SJS to SJS-TEN overlap syndrome to TEN. The hospitalization rate of SJS/TEN has gradually declined over time, but the mortality rate due to SJS/TEN has remained stable between 2010 and 2020.

We identified several risk factors associated with increased mortality including increasing age, racial/ethnic minority identification, chronic kidney disease, pneumonia, sepsis, and malignant neoplasm. This study lays the groundwork for future improvement of SJS/TEN mortality prediction scores/models such as SCORTEN. Based on this large cohort of patients with SJS/TEN, the SCORTEN model could be adjusted to improve prognostication by utilizing age as a continuous variable, by including chronic kidney disease, pneumonia, and sepsis as independent risk factors, and by including non-Hispanic White race/ethnicity as a proxy for reduced risk.

## Conflicts of interest

None disclosed.
